# Hypercalcemia As the Sole Initial Presentation of Precursor B-cell Acute Lymphoblastic Leukemia

**DOI:** 10.7759/cureus.22081

**Published:** 2022-02-10

**Authors:** Anup Kumar Trikannad Ashwini Kumar, Sruthi Vellanki, Geetha Krishnamoorthy

**Affiliations:** 1 Internal Medicine, Union Hospital, Terre Haute, USA; 2 Intenal Medicine, St. Joseph Mercy Oakland Hospital, Pontiac, USA

**Keywords:** leukemia, systemic chemotherapy, parathyroid hormone-related peptide (pthrp), acute lymphoblastic leukemia (all), acute hypercalcemia

## Abstract

A 24-year-old female presented with nausea, vomiting and abdominal pain. Physical examination was unremarkable. The patient's laboratory studies showed calcium of 17.2 mg/d, white cell count: 9,000/mcL with a normal peripheral blood smear. The patient had low PTH and PTHrp. She was hydrated, given calcitonin of four units/kg every 12 hours subcutaneously for 24 hours and zoledronate IV 4mg given once, with which calcium levels normalized and symptoms resolved. The patient returned one week later, with bone pain and bruises. Platelet count: 51,000/mcL, WBC count: 9,000/mcL, with lymphocytosis. A peripheral smear showed lymphoblasts. Flow cytometry confirmed precursor B-cell acute lymphoblastic leukemia (ALL) with 43% blasts. Hypercalcemic patients may have blasts at presentation, but can be “aleukemic.” Unexplained hypercalcemia with bone pain should lead to the suspicion of ALL, and a bone marrow exam should be performed even without peripheral blastosis to diagnose and treat ALL immediately.

## Introduction

Acute lymphoblastic leukemia (ALL) presents with neutropenic fever, bruising, anemia, lymphadenopathy, and bone pain. There are more cases reported of B-cell ALL associated hypercalcemia in children, but only a few have been found in young adults [1]. We report a rare case of a young adult female who presented with bone pain. An initial laboratory study showed elevated serum calcium which was appropriately treated. She was readmitted within a week due to ongoing severe bone pain. Repeat peripheral smear showed atypical cells highly suspicious for blasts and flow cytometry confirmed precursor B-cell ALL. She was transferred to a tertiary care center for treatment of ALL.

## Case presentation

A 24-year-old Caucasian female with a past medical history of irritable bowel syndrome, ovarian cyst and endometriosis presented to the emergency with vomiting, nausea, unintentional weight loss of 30 pounds over three weeks. The physical examination showed diffuse abdominal tenderness and no other signs including lymphadenopathy and hepatosplenomegaly. The patient denied any known family history of hematological disorders or anemias. She was admitted to our hospital and treated for unexplained severe symptomatic hypercalcemia; however, one week after discharge she presented with severe bone pain, mainly on her ribs lower back, multiple bruises and petechiae.

Investigation

**Figure 1:**
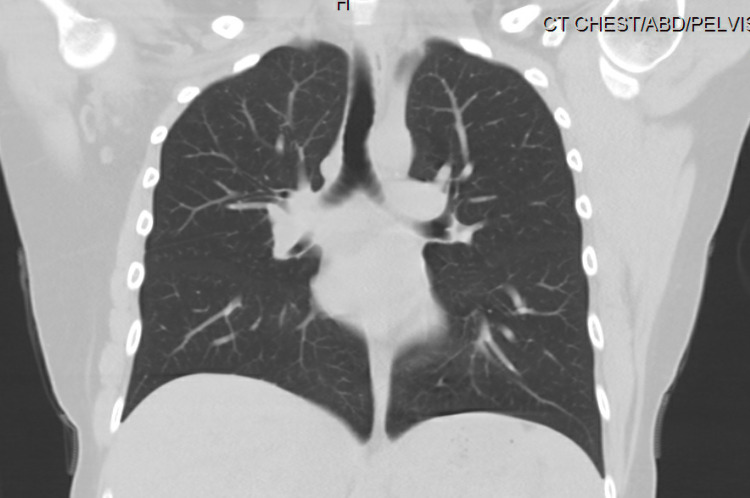
CT scan of the chest shows no evidence of mediastinal lymphadenopathy

**Figure 2:**
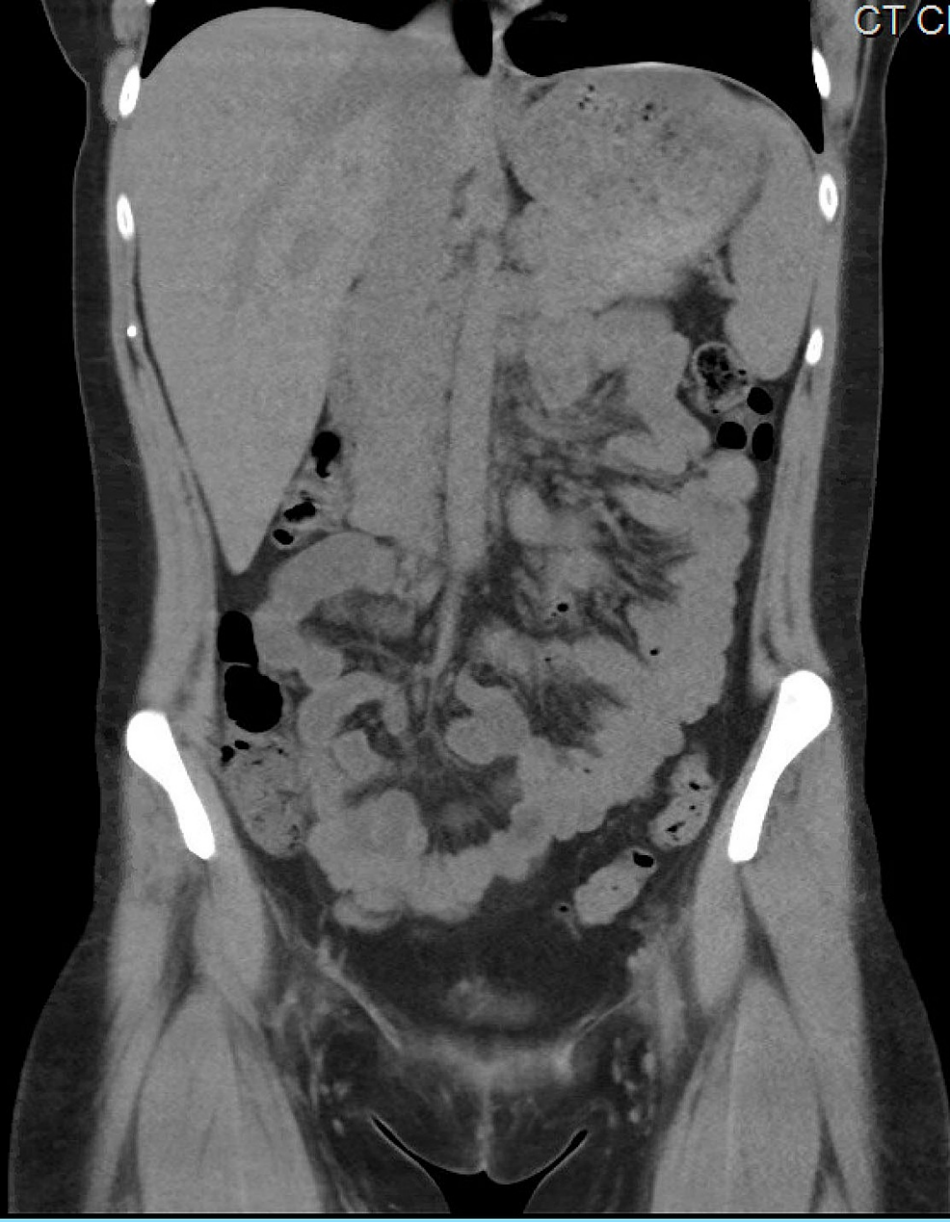
CT scan of the abdomen shows no masses to suggest abdominal malignancy

**Figure 3:**
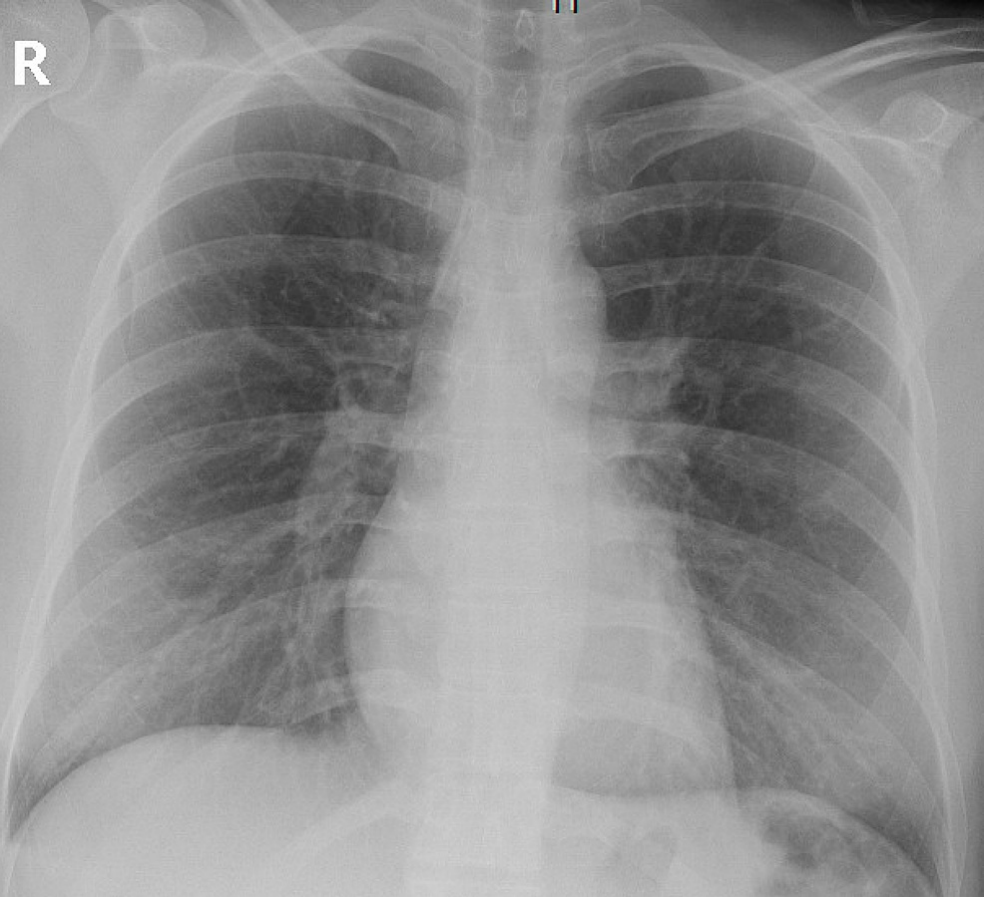
X-ray of the chest shows absence of osteolytic lesions on the ribs

**Figure 4:**
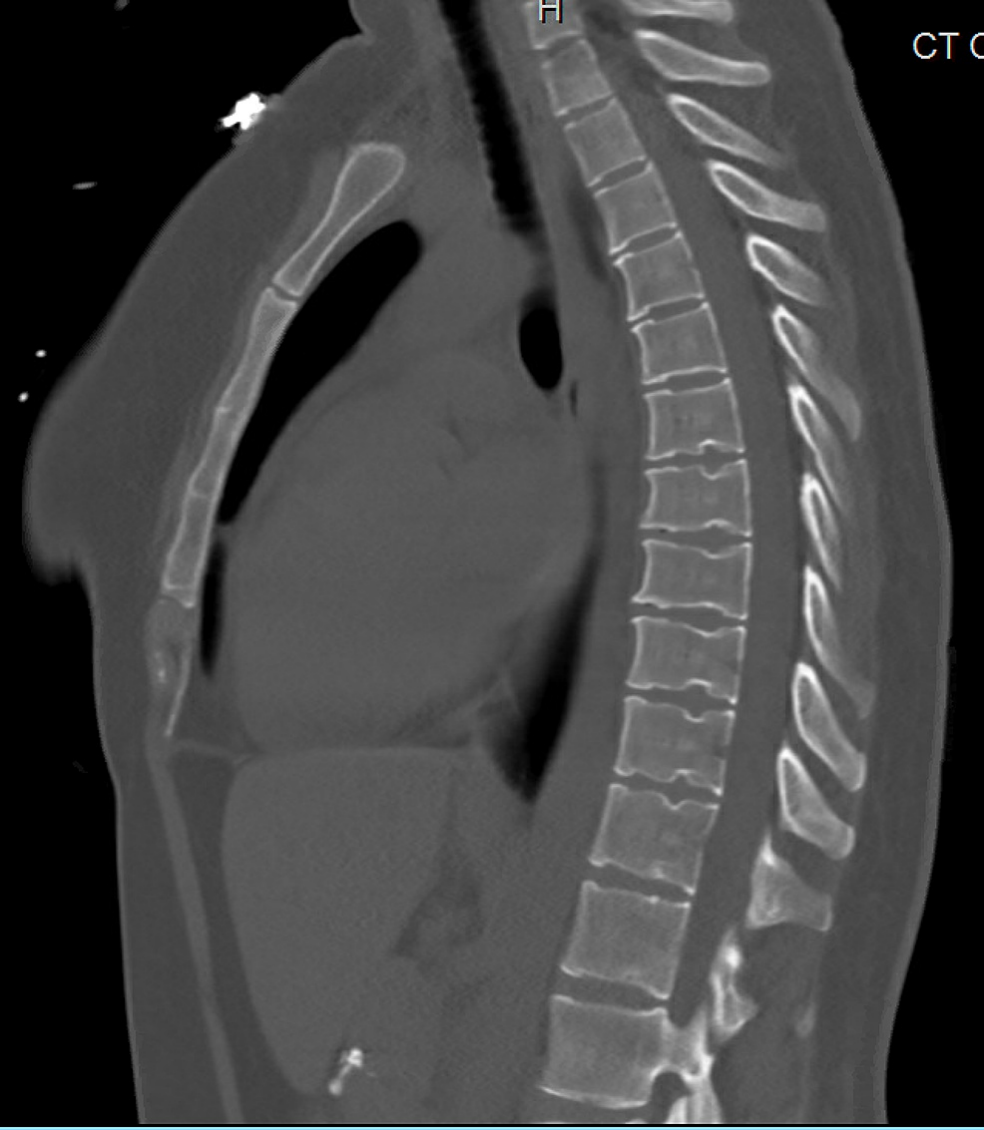
CT of the thoracic spine is not suggestive of osteolytic lesions

**Figure 5:**
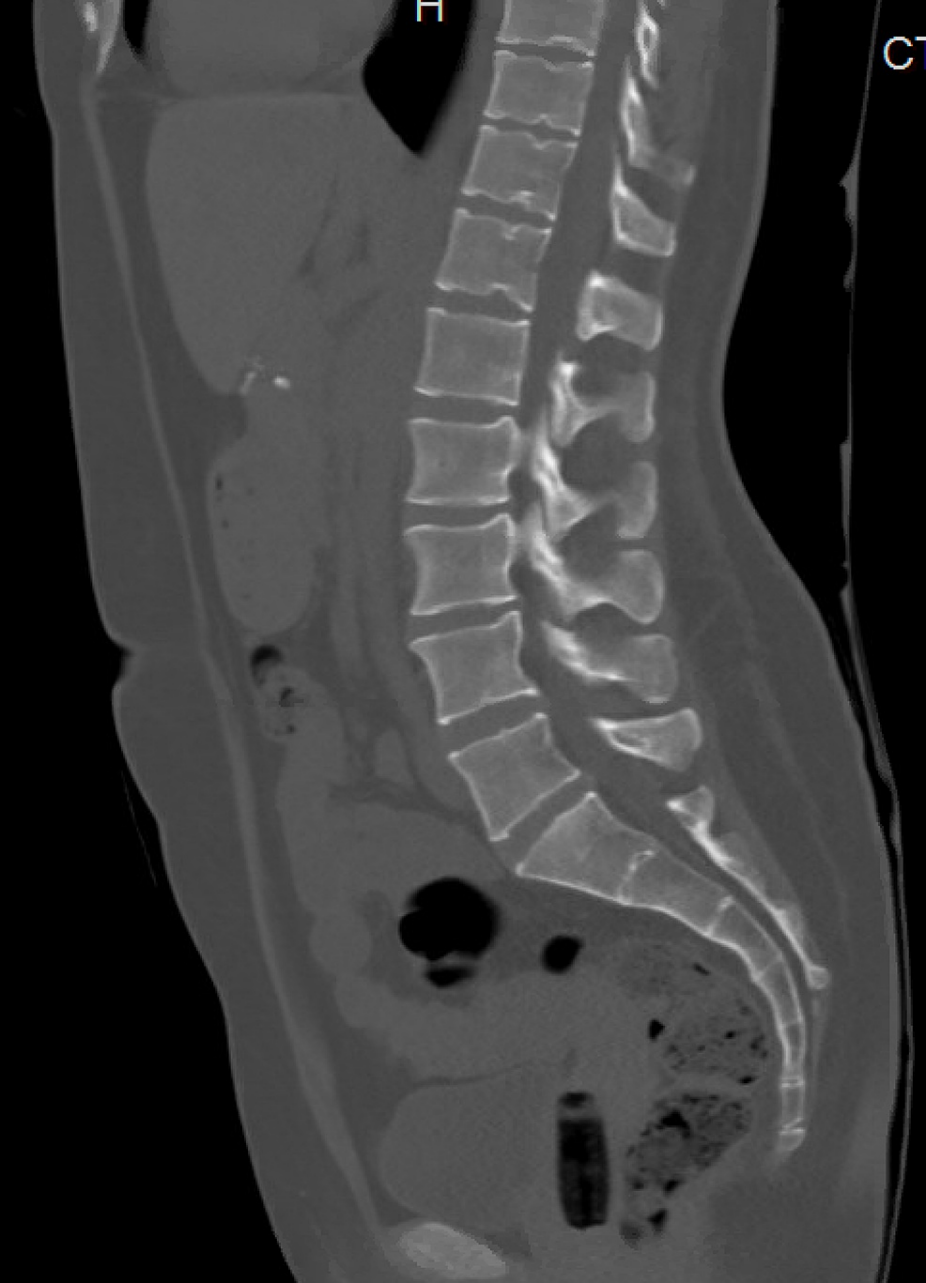
CT of the lumbar spine not suggestive of osteolytic lesions

Initial laboratory studies showed total calcium to be 17.2 mg/dL, ionized calcium 8.6 mg/dL, corrected calcium of 16.8 mg/dL, phosphorus 4.4 mg/dL, alkaline phosphatase 65 units per liter, albumin 4.5 g/dL, hemoglobin 15.5 g/dL, platelet count 136,000/mcL, absent atypical lymphocytes, metamyelocytes and absolute myelocytes. Absolute neutrophil count 8,300/mcL. In peripheral blood smear, atypical lymphocytes were noted suggestive of viral infection, moderate thrombocytopenia with no platelet clumping. CT of the chest and abdomen (Figures 1, 2) done to evaluate malignancy in the setting of hypercalcemia showed no evidence of malignancy. An X-ray of the chest and CT scan of the spine was obtained which showed no evidence of osteolytic lesions (Figures 3-5). No EKG changes were noted in spite of severe hypercalcemia. Ultrasound of pelvis and ultrasound transvaginal showed only minimal fluid in the posterior cul-de-sac and otherwise, the test was unremarkable. Serum levels of PTH were 3 pg per mL, vitamin D 25-hydroxy level 11.2 ng per mL. Vitamin D 1,25 dihydroxy was less than 5 pg per mL. Vitamin A level 26 mcg/dL. Parathyroid hormone-related protein 8 pg per mL, which was low. Immunofixation studies were normal and no M proteins were detected. The patient was admitted to the hospital for close monitoring and treatment of severe hypercalcemia. She was started on aggressive hydration with the initial bolus of 2 L followed by fluids at 200 mL per hour, then was given calcitonin 200 mg SubQ repeated once after 12 hours till the calcium returned to an acceptable range. In addition, she also received zoledronic acid 4 g intravenously. Five days into the stay in the hospital, calcium levels returned to the low normal of 7.5 mg/dL. PTH at the time of discharge was 132 pg per mL.

 

 

 

**Figure 6:**
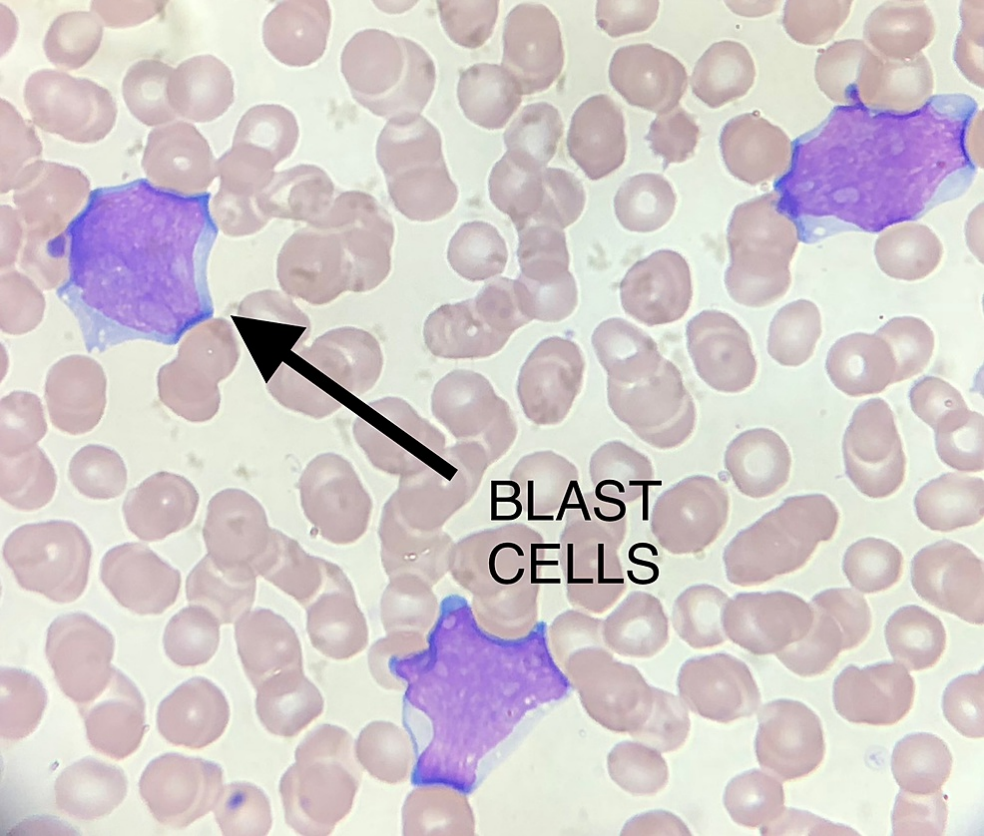
Peripheral smear shows the presence of blasts

The patient returned to the emergency room with worsening abdominal pain within a week and on repeat blood count, platelet count had dropped to 51,000/mcL, WBC count 9,000/mcL, neutrophil count 9 thou/mcL, absolute metamyelocyte count 0.09 thou/mcL, absolute myelocyte 0.18 thou/mcL. Peripheral smear showed normocytic, normocytic mild anemia. Normal absolute neutrophil count and absolute lymphocyte count with left shift with bands, metamyelocytes and myelocytes and no toxic reaction. Markedly atypical lymphocyte cells were present which was highly suspicious for blasts (Figure 6). Flow cytometric analysis was ordered and cytometry confirmed precursor B-cell ALL with a presence of 43% blasts.

Outcome and follow-up

The patient was transferred to tertiary care following diagnosis of B-cell ALL. She was started on treatment with R-hyper-CVAD and Dasatnib, which was later transitioned to Ponatinib. She completed her induction systemic chemotherapy as well as intrathecal chemotherapy. She is following outpatient for regular blood draws and monitoring.

## Discussion

Immediate intervention is required in hypercalcemia which is a common metabolic complication of malignant disease in adults. Hypercalcemia and osteolytic lesions as the presenting feature of ALL are documented in children, but very rare in adults [1]. Physicians need to be familiar with adult clinical manifestations of hypercalcemia and have to give consideration for blood diseases such as leukemias as a differential diagnosis as it is seen in a significant number of patients. The key complements of management of hypercalcemia are aggressive hydration and treatment of underlying malignancy. Hypercalcemia may occur as a presentation or transformation following a hematological neoplasm. The incidence of this type of cancer varies according to the type of cancer. Hypercalcemia as a complication of ALL has been reported to occur in only 2.5%-4.8% of patients [2].

Studies suggest cytokines including interleukin (IL)-2, IL-6, tumor necrosis, PGE2, and TGF-alpha might mediate hypercalcemia in ALL [3]. The pathogenesis of malignancy-associated hypercalcemia is not completely understood but is thought to be the result of local osteolytic metastases or enhanced bone resorption mediated by proteins and cytokines released by tumor cells [4]. Calcitriol has been associated with granulomatous disorders and certainly lymphomas and is implicated in hypercalcemia. PTH level elevation should prompt investigation for a parathyroid adenoma and ectopic PTHrP from solid malignancies. PTHrP produced by lymphoblasts causes hypercalcemia but Rizolli et all reported a lower prevalence of elevated PTHrP in hematopoietic malignancies [5]. The unregulated proliferation of lymphoblast can lead to an unchecked proliferation of proinflammatory cytokines that work as osteoclast activating factors.

In our workup hypercalcemia due to ectopic, PTHrP was ruled out as the mechanism causing bone reabsorption. Hypercalcemia secondary to vitamin D intoxication, primary hyperthyroidism, immobilization and solid malignancies were eliminated. Cytokines were not measured due to bioassay used for detection not being specific. Measurement of cytokines does not change management and it was a priority for antileukemic treatment since delay poses a life-threatening risk. An increase of proinflammatory cytokines seemed to be the most likely explanation for the hypercalcemia in our patient. Despite our patient having no blastosis on presentation, bone marrow biopsy revealed B-cell ALL.

## Conclusions

Our case demonstrates that ALL should be considered as a differential diagnosis in a young adult presenting with unexplained hypercalcemia in the absence of peripheral blasts cells as a rare presentation. Unexplained hypercalcemia with bone pain should lead to suspicion of ALL. Bone marrow should be performed early even without peripheral blastosis to diagnose and treat leukemia. Through this case we would Iike to highlight that inflammatory mediators such as tumor necrosis factors alpha, IL-6 and IL-2 can cause ALL-mediated hypercalcemia.
